# Impact of the COVID-19 pandemic on clinical and psychological aspects of temporomandibular disorders

**DOI:** 10.1186/s12903-024-04168-y

**Published:** 2024-04-12

**Authors:** Seong Hae Kim, Bo Young Jeong, Young Sun Kim, Ji Woon Park

**Affiliations:** 1 Center for Future Dentistry, School of Dentistry, Seoul National University, Seoul, Korea; 2 Dental Research Institute, Seoul National University, Seoul, Korea; 3 Department of Oral Medicine, Seoul National University Dental Hospital, Seoul, Korea; 4 Transdisciplinary Major in Learning Health Systems, Department of Public Health Sciences, Graduate School, Korea University, Seoul, Korea; 5 Department of Oral Medicine and Oral Diagnosis, Seoul National University School of Dentistry, 101 Daehak-ro, Jongno-gu, Seoul, 03080 Korea

**Keywords:** Temporomandibular disorders, Coronavirus, Pandemic, Pain, Psychological, Somatization

## Abstract

**Background:**

The Coronavirus 2019 disease (COVID-19) caused drastic changes in people’s lifestyle that affected TMD characteristics through its physical and psychological influences. The aim of this study was to define the clinical and psychological characteristics of a large group of well-defined TMD patients and seek their differences between before and during the COVID-19 pandemic to establish points of care to be emphasized in the post-pandemic era.

**Methods:**

TMD patients diagnosed by the Diagnostic Criteria for Temporomandibular Disorders (DC/TMD) aged ≥ 18 were analyzed. Samples between September, 2017 to July, 2019 (*n* = 455) and March, 2021 to June, 2022 (*n* = 338) were collected to represent before and during COVID-19, respectively. The Graded Chronic Pain Scale (GCPS) and Symptom Checklist-90-Revision (SCL-90-R) were used to evaluate disability levels and psychological status. Clinical indices were compared between COVID periods and factors related to higher pain levels were investigated according to pandemic period.

**Results:**

More patients reported pain on palpation of the masticatory muscles during the pandemic (*p* = 0.021) while the number decreased for neck muscles (*p* = 0.001) and TMJ (*p* < 0.001) areas. Patients reporting nocturnal bruxism (23.3–29.6%) and clenching (45.1–54.7%) significantly increased during the pandemic. TMD patients with pain without disability were more common during the pandemic regardless of pain intensity (*p* < 0.001). The number of patients expressing interference in daily activities decreased drastically during COVID-19 regardless of disability level (*p* < 0.001). Factors associated with higher than moderate pain intensity (CPI ≥ 50) were insomnia (odds ratio [OR] = 1.603, *p* = 0.047) and somatization (OR = 1.082, *p* < 0.001) before the pandemic. During the pandemic, age (OR = 1.024, *p* = 0.007), somatization (OR = 1.070, *p* = 0.006), and paranoid ideation (OR = 1.117, *p* = 0.003) were significantly associated with higher pain intensity.

**Conclusions:**

The results of our study underline the importance of evaluating psychological profiles of TMD patients, especially somatization, paranoid ideation and psychoticism, in exceptional situations that may cause a change in individual mental status. This will lead to a better understanding of the individual TMD patient and help in planning personalized treatment strategies that will assist the patient in adjusting to changes occurring in special environments such as the COVID-19 pandemic.

## Background

Temporomandibular disorders (TMD) is characterized by dysfunction and pain of the temporomandibular joints (TMJ), masticatory muscles, and surrounding structures. The prevalence of TMD in the general population has been reported as approximately 5–12% with women showing twice the risk compared to men [1, 2]. Although the etiology of TMD has not been clearly identified, previous research has shown that psychological factors such as anxiety and depression play a major role in its occurrence and prognosis [3]. Painful TMD showed increased associations with bronchitis and asthma as with widespread pain in adolescents. Also, those with painful TMD reported a higher number of systemic conditions compared to those free of TMD [4].

In November 2019, Coronavirus 2019 disease (COVID-19) broke out in Wuhan, China, rapidly spreading worldwide. The main symptoms of infected patients ranged from mild headaches to severe respiratory problems which led to death [5]. The World Health Organization (WHO) declared COVID-19 a pandemic in March, 2020 after which most governments implemented social distancing and partial to overall lockdown to prevent further spread of the virus. Decrease in social interactions with the rise of health threats, difficulty in accessing medical facilities, and income declines due to economic instability were among the many changes that affected people both physically and mentally. Not only did the majority of people fail to meet others and fulfill their social responsibilities, but also the fear of death and illness was prevalent [6, 7].

Studies reported that the incidence of musculoskeletal dysfunction and pain increased with COVID-19 infection [8] and 45.1% of patients still showed symptoms of musculoskeletal pain post-COVID recovery with de novo pain presenting in many [9]. Furthermore, research on orofacial pain conditions showed a high prevalence of TMD symptoms [10] and also twice the risk of TMD events in COVID-19 patients [11]. Recent research showed that chronic TMD patients with were more sensitive to distress caused by the pandemic and their psychological alterations resulted in increased orofacial pain levels [12]. As literature suggests, TMD is a complex disorder that is influenced by various biological, psychological, and social factors, all which were impacted by the pandemic condition and could have acted as health risks. Unfortunately, most related studies consisted of relatively small sample sizes and were based on results from online surveys and questionnaires.

Therefore, the aim of this study was to define the clinical and psychological characteristics of two large independent groups of well-defined TMD patients and seek their differences according to COVID-19 pandemic period to assess its influence on disease characteristics. Also, further analysis was done to investigate clinical indices that were closely related to TMD of higher pain levels in each pandemic period to define points of care to be emphasized in the post-pandemic era and their role in the biopsychosocial model of TMD.

## Materials and methods

### Subjects

Data was collected from consecutive “first-visit” patients over the age of 18 who were diagnosed by a single oral medicine specialist based on the Diagnostic Criteria for Temporomandibular Disorders (DC/TMD) between September, 2017 to June, 2022 [13].

A minimum of 105 patients per group was deemed necessary to attain a statistical power of 95% with an alpha error of 0.05 and a medium effect size for a two-tailed independent-samples t-test determined with G*Power software (version 3.1.9.7). A target sample size of 500 consecutive patients was set to enhance statistical accuracy and representativeness [14]. Finally, 455 and 338 patients each were included to represent the before (Sept 2017 to Jul 2019) and during COVID-19 (Mar 2021 to Jun 2022) period after excluding those with incomplete questionnaires and missing clinical information. The remaining numbers in each group were sufficient to allow adequate power of verification based on sample size estimation. The study was reviewed by the Institutional Review Board of Seoul National University Dental Hospital (reference number ERI22028). All subjects signed an informed consent form on their first visit approving the usage of their medical records for academic purposes. The need for obtaining further informed consent was waived by the Institutional Review Board based on the retrospective nature of the study.

### Assessment of clinical and psychological characteristics

Clinical indices including comfortable and maximum mouth opening range (mm), pain on palpation of the TMJ, masticatory and cervical muscle areas were recorded. The presence of tooth attrition, tongue, and buccal mucosa riding was verified through intraoral examinations. The Symptom Questionnaire (SQ) was used to collect comprehensive data on pain and related factors of TMD [13]. Parafunctional habits and oral behaviors were evaluated based on self-report.

The Graded Chronic Pain Scale (GCPS) version 2 was used to measure TMD pain severity and disability levels. Characteristic Pain Intensity (CPI) was calculated as the average of worst, present, and average pain intensity during the past month on a 0–10 visual analog scale (VAS) multiplied by 10. Interference level due to TMD pain was assessed with a 0–10 VAS.

Symptom Checklist-90-Revision (SCL-90-R), a self-report psychometric instrument, was applied for psychological evaluation [15].

### Statistical analysis

All analyses were performed after normality tests using Kolmogorov-Smirnov test. Independent t-test and Mann-Whitney U test was used for continuous variables and Chi-square test for discrete variables. Multiple regression and logistic regression analyses were done to determine variables associated with increased pain based on CPI scores. SPSS 25 (IBM SPSS Statistics Version 25.0. Armonk, NY: IBM Corp) was used in the analysis described above. For visualization of correlation analysis, ANACONDA (Anaconda, Inc., Texas, USA) was used with SPSS 25. Structural Equation Modeling (SEM) was used to evaluate the contribution of SCL-90-R sub-scores on GCPS variables. AMOS 26 (IBM AMOS Statistics Version 26.0. Armonk, NY: IBM Corp) was used to analyze the model. Before testing SEM, confirmatory factor analysis (CFA) was used to verify whether the single factor analysis (SFA) constructed as sub-items of the GCPS was suitable. An ideal model is determined when the average variance extracted (AVE) ≥ 0.5 [16] and the composite reliability (CR) ≥ 0.7 [17]. The reliability of the SFA model for before COVID-19 was AVE = 0942, CR = 0.980, and for during COVID-19 AVE = 0.882, CR = 0.957. With the latent variable in the center, each item of the SCL-90-R was used as a measurement variable on the left, and the measured GCPS variable was depicted on the right. Additionally, age and gender were added as independent variables to compensate for differences in variables based on the two items. Chi-square over degrees of freedom (χ^2^/DF), comparative fit index (CFI), root mean square error of approximation (RMSEA), goodness of fit index (GFI), and Tucker-Lewis index (TLI) were applied in determining the goodness of fit of SEM. The criteria for good fit were χ^2^/DF < 5, CFI, GFI, TLI > 0.9, RMSEA < 0.06 [18–20]. The level of significance was set α = 0.05 and a 95% confidence level.

## Results

### Clinical characteristics according to COVID-19 period

The average age of the total 793 participants was 37.6 years (14.9 standard deviation [SD]), of which 561 (70.7%) were women. As shown in Table 1, more patients reported pain on palpation of the masticatory muscles during the pandemic (*p* = 0.021) while the number decreased for neck muscles (*p* = 0.001) and TMJ (*p* < 0.001) areas. Patients showing TMJ noises, both click (*p* < 0.001) and crepitus (*p* < 0.001) increased during the pandemic. Joint locking showed a significant decrease (*p* < 0.001). Patients reporting nocturnal bruxism (23.3–29.6%) and clenching (45.1–54.7%) significantly increased during the pandemic.

**Table 1:** Clinical characteristics according to COVID-19 period

		Pre-COVID19 (*n* = 455)	During-COVID19 (*n* = 338)	Total (*n* = 793)	P-value
Age^§^		33.0 (27.0, 47.0)	32.0 (26.0, 45.0)	33.0 (26.0, 46.0)	0.155
Sex^‡^	Male	130 (28.6%)	102 (30.2%)	232 (29.3%)	0.623
	Female	325 (71.4%)	236 (69.8%)	561 (70.7%)	
Initial NRS^§^		4.0 (3.0, 6.0)	4.0 (2.0, 6.0)	4.0 (3.0, 6.0)	0.325
CMO (mm)^†^		39.3 (11.0)	39.0 (11.3)	39.2 (11.1)	0.719
MMO (mm)^†^		43.7 (9.5)	43.1 (9.7)	43.4 (9.6)	0.410
MOL (MMO ≤ 40)^‡^		125 (27.5%)	103 (30.5%)	228 (28.8%)	0.356
Pain on mouth opening^‡^		223 (49.0%)	183 (54.1%)	406 (51.2%)	0.153
Palpation^‡^	Masticatory muscles	255 (56.0%)	217 (64.2%)	472 (59.5%)	**0.021** ^*^
	Neck muscles	141 (31.0%)	69 (20.4%)	210 (26.5%)	**0.001** ^*^
	TMJ	167 (36.7%)	79 (23.4%)	246 (31.0%)	**< 0.001** ^*^
Sound^‡^	Click	103 (22.6%)	167 (49.4%)	270 (34.0%)	**< 0.001** ^*^
	Crepitus	38 (8.4%)	62 (18.3%)	100 (12.6%)	**< 0.001** ^*^
Joint locking^‡^		118 (25.9%)	13 (3.8%)	131 (16.5%)	**< 0.001** ^*^
Tooth attrition^‡^		166 (36.5%)	77 (22.8%)	243 (30.6%)	**< 0.001** ^*^
Tongue ridging^‡^		242 (53.2%)	164 (48.5%)	406 (51.2%)	0.194
Mucosal ridging^‡^		305 (67.0%)	193 (57.1%)	498 (62.8%)	**0.004** ^*^
Contributing factors^‡^	Bruxism	106 (23.3%)	100 (29.6%)	206 (26.0%)	**0.046** ^*^
	Clenching	205 (45.1%)	185 (54.7%)	390 (49.2%)	**0.007** ^*^
	Perioral contraction	63 (13.8%)	46 (13.6%)	109 (13.7%)	0.924
	Tongue thrusting	27 (5.9%)	24 (7.1%)	51 (6.4%)	0.508
	Insomnia	135 (29.7%)	89 (26.3%)	224 (28.2%)	0.302

NRS, numerical rating scale; CMO, Comfortable mouth opening; MMO, maximum mouth opening; MOL, mouth opening limitation; TMJ, temporomandibular joint

^†^Differences between groups were tested with Independent T-test: mean (SD)

^‡^Differences between groups were tested with chi-square test: number of subjects or positive answers (%)

^§^Differences between groups were tested with Mann-Whitney U test: median (25%, 75%)

^*^Significant difference, *p* < 0.05

### Disability level and psychological conditions according to COVID period

As shown in Table 2, TMD patients with pain without disability were more common during the pandemic regardless of pain intensity (*p* < 0.001). The number of patients expressing interference in daily activities decreased drastically during COVID-19 regardless of disability level (*p* < 0.001). The level of current pain showed a significant decrease in mean (3.4 to 3.0). Interference level and days experiencing interference all decreased during COVID-19. The results of SCL-90-R are shown in Table 3. Depression was the dimension with most patients showing high scores indicating pathologic status both before and during the pandemic.

**Table 2:** Pain and disability level according to COVID-19 period

			Pre-COVID19 (*n* = 455)	During-COVID19 (*n* = 338)	Total (*n* = 793)	P-value
Grade^†^	No disability (0)		72 (15.8%)	46 (13.6%)	118 (14.9%)	**< 0.001** ^*^
	Low intensity pain without disability (I)		121 (26.6%)	155 (45.9%)	276 (34.8%)	
	High intensity pain without disability (II)		66 (14.5%)	103 (30.5%)	169 (21.3%)	
	Moderately limiting (III)		120 (26.4%)	29 (8.6%)	149 (18.8%)	
	Severely limiting (IV)		76 (16.7%)	5 (1.5%)	81 (10.2%)	
CPI^‡^			40.0 (20.0, 60.0)	40.0 (19.3, 57.0)	40.0 (20.0, 60.0)	0.399
CPI ≥ 50^†^			186 (40.9%)	124 (36.7%)	310 (39.1%)	0.231
Current pain^‡^			3.0 (1.0, 5.0)	3.0 (1.0, 5.0)	3.0 (1.0, 5.0)	**0.025** ^*^
Worst pain^‡^			5.0 (2.0, 7.0)	5.0 (2.8, 7.0)	5.0 (2.0, 7.0)	0.403
Average pain^‡^			4.0 (1.0, 6.0)	3.0 (1.0, 5.0)	3.0 (1.0, 6.0)	0.243
Number of days of pain^‡^			15.0 (3.0, 51.0)	15.0 (3.0, 50.0)	15.0 (3.0, 50.0)	0.877
Interference level		Daily activity^‡^	3.0 (1.0, 5.0)	2.0 (0.0, 5.0)	3.0 (0.0, 5.0)	**0.001** ^*^
		Social^‡^	3.0 (0.0, 5.0)	2.0 (0.0, 4.0)	2.0 (0.0, 5.0)	**< 0.001** ^*^
		Work activity^‡^	3.0 (0.0, 5.0)	1.0 (0.0, 4.0)	2.0 (0.0, 5.0)	**< 0.001** ^*^
Number of days with interference^‡^			7.0 (0.0, 30.0)	0.0 (0.0, 1.0)	0.0 (0.0, 15.0)	**< 0.001** ^*^

CPI, characteristic pain intensity

^†^ Differences between groups were tested with chi-square test: number of subjects or positive answers (%)

^‡^ Differences between groups were tested with Mann-Whitney U test: median (25%, 75%)

^*^ Significant difference, *p* < 0.05

**Table 3:** Psychological characteristics according to COVID-19 period

	Pre-COVID19 (*n* = 455)	During-COVID19 (*n* = 338)	Total (*n* = 793)	P-value
Depression^†^	40.0 (36.0, 47.0)	41.0 (37.0, 48.0)	41.0 (36.0, 48.0)	0.387
Anxiety^†^	40.0 (38.0, 46.0)	40.0 (38.0, 46.0)	40.0 (38.0, 46.0)	0.681
Somatization^†^	43.0 (39.0, 48.0)	43.0 (40.0, 48.0)	43.0 (40.0, 48.0)	0.918
Obsessive-compulsive^‡^	42.6 (8.9)	42.4 (8.5)	42.5 (8.7)	0.735
Interpersonal sensitivity^†^	41.0 (36.0, 46.0)	41.0 (37.0, 46.0)	41.0 (37.0, 46.0)	0.279
Hostility^†^	40.0 (40.0, 45.0)	42.0 (40.0, 45.0)	40.0 (40.0, 45.0)	0.131
Phobic anxiety^†^	42.0 (40.0, 45.0)	42.0 (40.0, 45.0)	42.0 (40.0, 45.0)	0.990
Paranoid ideation^†^	38.0 (38.0, 42.0)	39.5 (38.0 45.0)	38.0 (38.0, 42.0)	0.104
Psychoticism^†^	40.0 (38.0, 44.0)	40.0 (38.7, 45.0)	40.0 (38.0, 45.0)	0.362

^†^ Differences between groups were tested with Mann-Whitney U test: median (25%, 75%).

^‡^ Differences between groups were tested with Independent T-test: mean (SD).

^*^ Significant difference, *p* < 0.05.

### Correlations among clinical and psychological variables according to COVID period

**Fig. 1:**
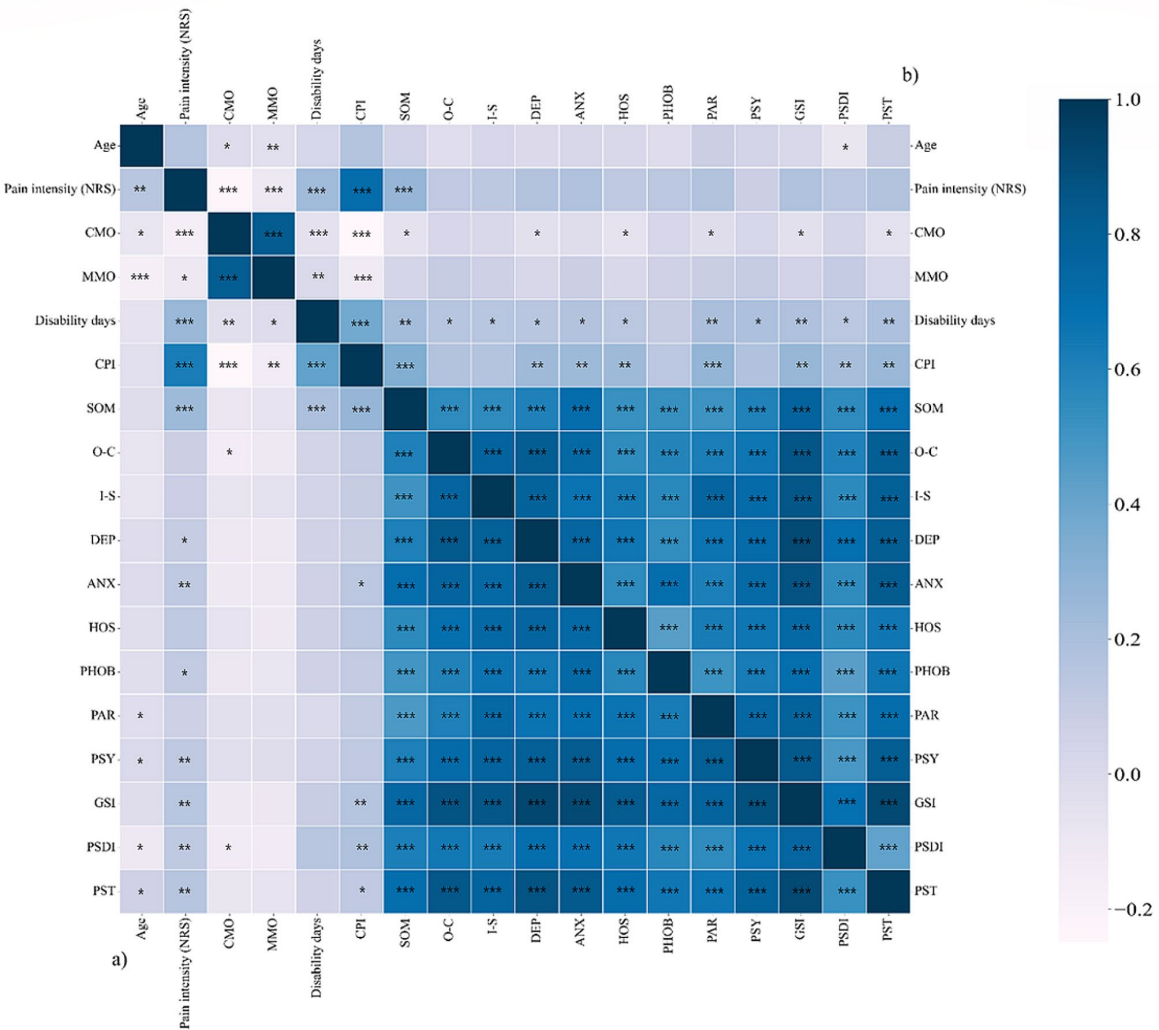
Heatmap showing the correlation between SCL-90-R sub-dimensions and clinical characteristics. Based on the diagonal line where the correlation appears at 1.0, the lower triangle is (**a**) Before COVID-19 and the upper triangle is (**b**) During COVID-19. NRS, numerical rating scale; CMO, comfortable mouth opening; MMO, maxium mouth opening; CPI, characteristic pain intensity; SOM, somatization; O-C, obsessive-compulsive; I-S, interpersonal sensitivity; DEP, depression; ANX, anxiety; HOS, hostility; PHOB, phobic anxiety; PAR, paranoid ideation; PSY, psychoticism; PST, positive symptom total; PSDI, positive symptom distress index; GSI, global severity index. *, *p* < 0.05; **,*p* < 0.01; ***, *p* < 0.001

Figure 1 shows results from Spearman’s correlation analysis as heatmap with darker blue indicating higher correlation (coefficient closer to -1.0 or 1.0) and whiter a coefficient closer to 0. Before the pandemic, somatization scores from SCL-90-R showed a significant correlation with pain intensity based on a 0–10 numeric rating scale (NRS) (*r* = 0.239, *p* < 0.001), disability days (*r* = 0.205, *p* < 0.001), and CPI (*r* = 0.267, *p* < 0.001). During the pandemic, somatization scores showed significant correlation with CPI (*r* = 0.269, *p* < 0.001) and pain intensity NRS (*r* = 0.214, *p* < 0.001). Unlike before the pandemic, paranoid ideation also showed significant correlation with CPI (*r* = 0.203, *p* < 0.001).

### Factors related to higher TMD pain levels according to COVID period

Multiple regression analysis was conducted using clinical and psychological variables to determine their effect on pain intensity based on CPI. Somatization was found to be the only variable showing a consistent positive relationship with pain intensity in both periods (*p* < 0.001).

Results from logistic regression analysis are shown in Table 4. Factors associated with higher than moderate pain intensity (CPI ≥ 50) were insomnia (odds ratio [OR] = 1.608, *p* = 0.041) and somatization (OR = 1.080, *p* < 0.001) before the pandemic. During the pandemic, age (OR = 1.020, *p* = 0.023), somatization (OR = 1.072, *p* = 0.004), and paranoid ideation (OR = 1.121, *p* = 0.002) were significantly associated with higher pain intensity.

**Table 4:** Psychological indices associated with moderate to severe pain according to COVID-19 period

	Variable	OR	95% CI		p-value
			Lower	Upper	
Pre- COVID19 (*n* = 455)	Age	1.005	0.992	1.018	0.453
	Sex^†^	1.480	0.931	2.351	0.097
	Insomnia	1.608	1.019	2.537	**0.041** ^*^
	Depression	0.940	0.893	0.991	**0.020** ^*^
	Anxiety	0.980	0.923	1.040	0.503
	Somatization	1.080	1.041	1.120	**< 0.001** ^*^
	Interpersonal Sensitivity	1.016	0.972	1.063	0.486
	Paranoid Ideation	0.977	0.926	1.031	0.400
	Psychoticism	1.058	0.982	1.139	0.139
During-COVID19 (*n* = 338)	Age	1.020	1.003	1.037	**0.023** ^*^
	Sex^†^	0.886	0.509	1.541	0.668
	Insomnia	0.920	0.511	1.657	0.782
	Depression	1.062	0.997	1.132	0.060
	Anxiety	1.027	0.959	1.099	0.452
	Somatization	1.072	1.023	1.123	**0.004** ^*^
	Interpersonal Sensitivity	0.947	0.891	1.006	0.078
	Paranoid Ideation	1.121	1.043	1.204	**0.002** ^*^
	Psychoticism	0.903	0.831	0.982	**0.018** ^*^

The results were obtained from logistic regression analysis

Moderate to severe pain: characteristic pain intensity ≥ 50

^†^Reference group for statistical comparisons: Sex = male and contributing factors = negative

^*^Significant difference: *p* < 0.05

**Fig. 2:**
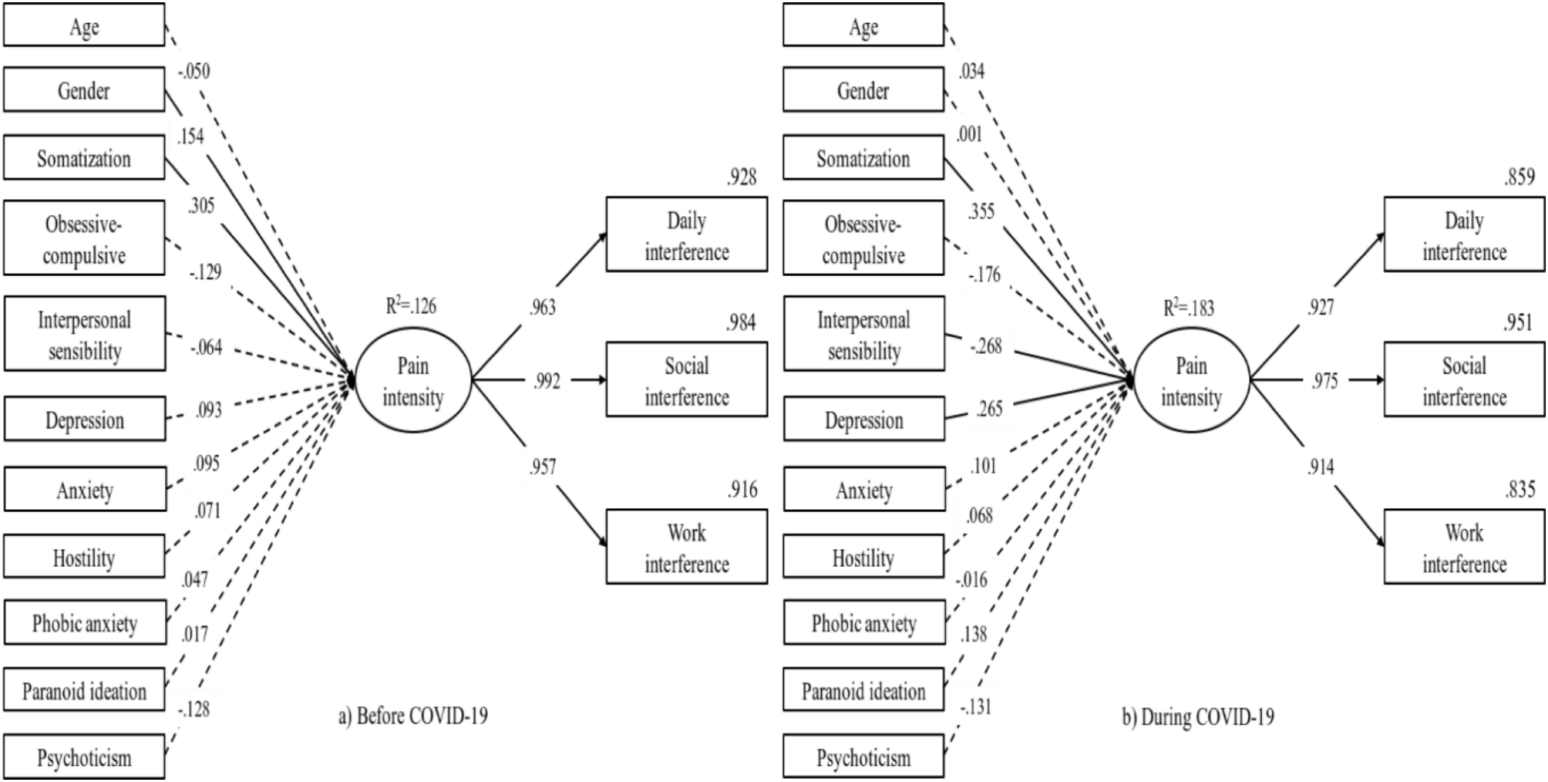
Structural equation model of psychological characteristics associated with pain intensity leading to functional interference before and during the COVID-19 pandemic. Variables that have statistically significant effects are connected by solid lines, while insignificant variables are connected by dotted lines

The results of SEM analysis are shown in Fig. 2. Both before COVID-19 (chi-square = 37.225; DF = 22; CMIN/DF = 1.692; GFI = 0.989; TLI = 0.990; CFI = 0.998; RMSEA = 0.039 [0.015 − 0.060]) and during COVID-19 model (chi-square = 36.131; DF = 22; CMIN/DF = 1.642; GFI = 0.986; TLI = 0.983; CFI = 0.996; RMSEA = 0.044 [0.014 − 0.068]) showed good fit. Somatization (*p* < 0.001) significantly affected pain intensity based on GCPS before COVID-19. During COVID-19, somatization (*p* < 0.001), interpersonal sensitivity (*p* = 0.006), and depression (*p* = 0.012) significantly affected pain intensity. In both periods, the effect of each item of SCL-90-R on pain intensity was low with the highest factor loading of 0.36.

## Discussion

The main goal of this retrospective study was to compare clinical characteristics of TMD patients before and during the pandemic and to investigate risk factors associated with higher pain intensity according to COVID-19 period. The results of this study showed that there were changes in clinical characteristics of TMD during the COVID-19 pandemic. More patients reported pain on palpation of the masticatory muscles while fewer patients reported pain on TMJ area palpation during the pandemic. At the same time, more patients reported the presence of nocturnal bruxism and tooth clenching. Those experiencing pain regardless of intensity increased during the pandemic while those reporting disability due to TMD decreased. Different psychological conditions were associated with moderate to severe TMD pain during the pandemic indicating a change in the influence of psychological factors on TMD pain characteristics.

The patients who sought care during the lockdown presented more masticatory muscle pain, TMJ sounds, and self-reported parafunctional habits than those who attended before the pandemic. These findings were corroborated by aggravation of TMD symptoms and oral parafunction reported during the COVID-19 pandemic in previous literature [21]. Patients showed greater rates of myalgia, headache, and degenerative joint disease during the pandemic than those who visited before. They also had significantly more pain-related and intra-articular TMD conditions [22]. A study on rheumatoid arthritis patients also reported a significant increase in the prevalence of fibromyalgia and disease activity during the pandemic compared to pre-COVID-19 periods [23]. However, the results of our analysis showed that fewer patients reported pain on TMJ and cervical muscle palpation during the pandemic. The consequences of COVID-19-related musculoskeletal pain symptoms are not yet fully understood and diverse physiological processes have been proposed to be associated with a wide range of clinical symptoms [24]. Our result is congruous to an Italian study showing that the prevalence of TMD pain and joint sounds incremented in correlation with gender, age, and stress level during the pandemic [25]. TMJ noise is the most common symptom in TMD patients. However, its intensity does not show a direct positive association with pain nor functional limitation levels of TMD. TMJ noises may be identified as an early sign of TMD and as the disease progresses into degenerative joint disease [26]. Previous studies have shown that patients with oral parafunctions had a higher prevalence of TMJ noise compared to those without [27]. Studies have also presented a correlation between nocturnal bruxism and the diagnosis of TMD [28]. Parafunctional habits are considered to be involved in peripheral mechanisms which are likely to play a part in TMD onset while the chronification of TMD symptoms is more likely to depend on central components including sensitization and genetic factors. Bruxism is generally considered as innocuous unless the forces exerted in addition to other aggravating factors outpace physiological resilience and result in elevated risk of structural change and deep pain input [29]. As the global health emergency had been ongoing for three years, people’s mental stress has increased due to concerns about infection and social distancing. Since mental stress is known to have a negative effect on oral parafunctions the significant increase of bruxism observed in our TMD patients during the COVID-19 outbreak could be a direct result of deteriorating mental health. Accumulated psychological distress can induce sympathetic activity which leads to the release of endogenous steroids, promoting vasoconstriction of muscles and increase in peripheral vascular resistance [30]. Increased sympathetic signals and hyperarousal can account for sleep deprivation as well, which may heighten mental stress levels to create a vicious cycle [31]. Since oral parafunction was not investigated objectively the increased level of parafunctional habits observed in this study may be interpreted as a result of an increase in patient awareness during the daytime. Psychosocial factors including perceived stress, depression, and anxiety have been proposed to generate awake bruxism [32, 33].

Those experiencing pain regardless of intensity (GCPS grade 1,2) increased during the pandemic while those who answered that pain hinders their lives decreased significantly. These results are supported by other papers that have explored the impact of pain experiences and mood disasters during COVID-19. Adjusted coping strategies including increased exercise in the lockdown period could lower pain severity and interference level [34]. Another prior study depicted that the social disability domain improved post-pandemic however, the results were limited to female TMD patients [7]. On the other hand, there are studies that report increase in pain interference in the majority of a pain patient group with social distancing [35]. Studies on pain interference during the pandemic based on GCPS are limited, so it is difficult to directly compare our results with those based on different assessment tools. Fewer occupational activities and increased work-from-home status during the pandemic may be related to the improvement in pain interference scores of our study. Another research concluded that the COVID-19 situation did not affect pain intensity or health-related quality of life emphasizing the importance of individual skills in handling chronic pain [36]. In case of certain conditions such as endometriosis, chronic pain symptoms and global physical impairment even improved post lockdown [37].

Contrary to a recent review reporting a high prevalence of negative psychiatric symptoms during the pandemic [38] and several studies showing COVID-19 to have a considerable negative impact on emotional status which prompted an exacerbation of bruxism and TMD symptoms [39] no significant change was found in our TMD patients before and during COVID-19. The current investigation found a difference in psychological characteristics associated with moderate to severe pain before (depression and somatization) and during (paranoid ideation, psychoticism, and somatization) the pandemic. Somatization consistently predicted painful TMD in both periods. Patients with painful TMD often exhibit moderate to high levels of somatization [40, 41]. Somatization is more prevalent in TMD patients compared to the general population [42]. Also, it is known to predict chronic and widespread pain even without organic disease [43]. Somatization was more prevalent among bruxers and was the only variable that demonstrated a significant correlation with the diagnosis of myofascial pain [44]. Another study reported the association between severe somatization and high interference levels [45]. The positive correlation between pain intensity and paranoid ideation post COVID-19 lockdown might imply pain catastrophizing or maladaptive cognitive measures implemented by patients suffering from moderate to severe pain [46]. The overall results of our study showing increase in those reporting masticatory muscle pain and the consistent association of somatization with moderate to severe pain underline the need of both biological and psychosocial methods to be applied in the evaluation and treatment of TMD for best results. And cardinal psychological and social aspects of TMD are prone to change depending on the presence of specific surrounding conditions [47].

There are several limitations to consider when interpreting the results. All patients in this present study consisted of South Koreans and specific ethnic and cultural factors may have influenced the results and limited its generalizability. Further research comparing results from different countries and ethnic backgrounds may provide valuable data on the impact of the COVID-19 pandemic on TMD characteristics. Secondly, due to the retrospective nature of the study, possibilities of sampling bias exist. However, the total sample size is relatively large compared to other studies on TMD and the patient group was defined based on a well-standardized diagnostic process supporting the reliability of the results. Still, the OR for factors associated with higher than moderate intensity pain are relatively small indicating weak association. Also, investigations were conducted only for a limited period of time during the COVID-19 pandemic. Future studies should be designed to include a longer research period which would have allowed better understanding of the longitudinal impact of the ongoing COVID-19 pandemic. Thirdly, some clinical parameters relied on self-report thus, lacking objectivity. Further research including psychological assessment done by trained experts are needed to elucidate the correlation between pain intensity and mental health in TMD under pandemic conditions. Finally, the study consisted of two independent cohorts of patients from before and during the pandemic analyzed each in a cross-sectional manner. The study design does not allow the evaluation of causation but only differences between the cohorts due to COVID-19. To identify causality, future studies should follow the change in disease characteristics of an identical group of patients before and after the pandemic.

## Conclusion

TMD symptoms including masticatory muscle pain, TMJ noise, and parafunctional habits increased during the pandemic compared to pre-COVID-19. On the other hand, subjective pain interference decreased, which implies the importance of individual coping strategies. Somatization emerged as the sole predictor of pain intensity both before and during the pandemic while different psychological aspects were associated with moderate to severe TMD pain according to the pandemic period. Mental health affects individual coping and management strategies in adverse times. The results of our study underline the importance of evaluating psychological profiles, especially somatization, paranoid ideation and psychoticism, of TMD patients in exceptional situations that may cause a change in individual mental status. This will lead to a better understanding of the individual TMD patient and help in planning personalized treatment strategies that will assist the patient in adjusting to the changes occurring in special environments such as the COVID-19 pandemic.

## Data Availability

The raw data supporting this study are not in the public domain but are available upon reasonable request from the corresponding author.

## Abbreviations

TMD: Temporomandibular disorders
TMJ: temporomandibular joints
COVID-19: coronavirus 2019 disease
DC/TMD: diagnostic criteria for temporomandibular disorders
SQ: symptom questionnaire
WHO: World Health organization
GCPS: graded chronic pain scale
CPI: characteristic pain intensity
VAS: visual analog scale
SCL-90-R: symptom checklist-90-revision
PST: positive symptom total
GSI: global severity index
PSDI: positive symptom distress index
SEM: structural equation modeling
CFA: confirmatory factor analysis
SFA: single factor analysis
AVE: average variance extracted
CR: composite reliability
χ^2^/DF: Chi-square over degrees of freedom
CFI: comparative fit index
RMSEA: root mean square error of approximation
GFI: goodness of fit index
TLI: Tucker-Lewis index
SD: standard deviation
NRS: numeric rating scale
OR: odds ratio
CMO: comfortable mouth opening
MMO: maximum mouth opening
MOL: mouth opening limitation
SOM: somatization
O-C: obsessive-compulsive
I-S: interpersonal sensitivity
DEP: depression
ANX: anxiety
HOS: hostility
PHOB: phobic anxiety
PAR: paranoid ideation
PSY: psychoticism
